# A personalized multi-interventional approach focusing on customized nutrition, progressive fitness, and lifestyle modification resulted in the reduction of HbA1c, fasting blood sugar and weight in type 2 diabetes: a retrospective study

**DOI:** 10.1186/s12902-022-01212-2

**Published:** 2022-11-22

**Authors:** Chhavi Goyal Mehra, Annie Mattilda Raymond, Rekha Prabhu

**Affiliations:** Ragus Healthcare Pvt Ltd, Sugar.Fit, HSR Layout, Sector 3, Bengaluru, Karnataka 560102 India

**Keywords:** Type 2 diabetes reversal, Nutrition, Fitness, Lifestyle modification, HbA1c reduction, Fasting blood sugar, Weight loss

## Abstract

**Background:**

Type 2 diabetes (T2D) is a chronic, progressive lifestyle disease and the most rapidly growing health challenge of the twenty-first century. The American Diabetes Association recommends that T2D reversal can be achieved through an organized, and systematic approach focusing on nutrition, fitness, and lifestyle management.

**Aim:**

This study aimed to evaluate the effectiveness of a comprehensive and multi-interventional diabetes care program called Sugar. Fit Diabetes Reversal Programme (SDRP) on glycosylated haemoglobin (HbA1c), fasting blood sugar (FBS), and body weight for T2D reversal.

**Methodology:**

SDRP is a personalized intervention study that uses technology-enabled medical management, dedicated coach-led diabetes, and nutrition experts. The study involved 150 patients living with type 2 diabetes in the age group of 20 to 80 years and having HbA1c of > 6.5%. In SDRP, the participants were assigned personal medical doctors specializing in diabetes, along with health coaches for providing customized nutrition, personalized fitness routines, relevant lifestyle modifications to holistically reverse type 2 diabetes. The HbA1c level, fasting blood sugar, and weight of the participants were measured at baseline and the end of the study (90th day). The effectiveness of SDRP was analyzed by comparing it with a control group that involved 110 individuals with type 2 diabetes managed by conventional pharmacotherapy and regular dietary advice but not participating in the SDRP.

**Results:**

All 150 participants adhered to the program for 90 days. The analysis was performed on participants and represented as mean ± standard deviation (mean ± SD). At the end of SDRP, a significant reduction in HbA1c level, FBS, and weight was observed as compared to the control group. The results showed that Hba1c levels dropped from 9.0 ± 1.5% to 7.1 ± 1.3% with a mean change of 1.9 ± 1.5%; FBS levels decreased from 178.3 ± 57.1 mg/dL to 116.1 ± 24.2 mg/dL with a mean loss of 62.2 ± 51.8 mg/dL, and the weight decreased from 76.7 ± 12.7 kg to 73.8 ± 11.8 kg with a mean weight loss of 2.8 ± 1.6 kg. The results also showed that participants between 20 to 35 years showed the highest drop in HbA1c, FBS, and weight.

**Conclusion:**

The findings indicate that a comprehensive and multi-interventional diabetes care program involving personalized nutrition, fitness, and lifestyle modification such as SDRP, help in significant and sustained improvements in HbA1c level, glycaemic control, and weight loss in adults with type 2 diabetes.

## Introduction

In 2017, International Diabetes Federation (IDF) reported 425 million people worldwide to have diabetes. This number is expected to go up to 550 million by the year 2030 [1–3]. Diabetes is the major epidemic of this century; in 2019, 1.5million deaths occurred directly from diabetes, not counting deaths from complications of diabetes. Uncontrolled Type 2 diabetes leads to higher doses and number of anti glycemic meds, higher rates of hospital admissions secondary to complications, and high mortality rates; this is creating a significant burden on healthcare costs [4]. Medical interventions do not alone seem to be able to reverse or improve outcomes in diabetes. Slowing the progression and aiming to reverse by sustainable lifestyle interventions alone or, in some cases, medication can lead to beta cell recuperation and metabolic recovery, which seems promising for long-term remission post reversal [4, 5].

The World Health Organization (WHO) global diabetes report 2016 has been clearly stated that diabetes reversal can be achieved by calorie restriction and weight loss. This validation by WHO has changed the earlier paradigm of diabetes from being chronic and irreversible. Maintaining haemoglobin A1c (HbA1c) below the diabetic threshold of 6.5% for a prolonged duration without using glycemic control medications except metformin will be considered diabetes reversal or remission [6, 7].

In the past years, various approaches were adapted for achieving reversal of diabetes, namely, the standard of care and the status quo approach, bariatric surgery [8], low-calorie diets, or carbohydrate restriction, and nutritional ketosis. Though these approaches were reported to be effective in reversing diabetes through weight loss and improvements in glycemic control, they have unavoidable limitations. An extensive study reported that diabetes remission is rare and variable with standard of care and the status quo approach [5]. While bariatric surgery is expensive, post-operative weight gain and the possible risk of nutritional deficiencies limit its use. Further, low-calorie diets, carbohydrate restrictions, and nutritional ketosis are not sustainable as adhering to the weight loss diet and maintenance is a persisting challenge [5]. Thus, there is a need for a feasible, sustainable, long-term, and effective diabetes reversal or remission approach.

According to the American Diabetes Association (ADA), an ideal approach for diabetes management should be an organized, systematic, and individualized patient-centred lifestyle management. ADA suggests that the approaches should include diabetes self-management education and support, which can be given in a group or individually or using technology. Approaches such as nutrition therapy and physical activity should support healthy eating patterns to achieve and maintain body weight, glycemic index, blood pressure, and lipid goals and dictate the frequency and timing for self-monitoring of blood glucose levels [9].

Since diabetes care is chronic and requires sustainable behavioural change, adhering to lifestyle changes could be infrequent and difficult without the proper support from a health care provider and peer. Thus we propose a comprehensive diabetes care program that supports the people with T2D to achieve diabetes reversal. We call this multi-interventional approach as “Sugar.fit Diabetes Reversal Programme (SDRP).”

SDRP is a deep tech-enabled coach lead program with diabetes expert physicians and a specialized nutritionist generating highly personalized lifestyle interventions like meal plans, progressive fitness plans, and behavioural modifications.

This study aimed to evaluate the effectiveness of SDRP on glycosylated haemoglobin (HbA1c), fasting blood sugar (FBS), and body weight for T2D reversal or remission. Patient age, gender, weight, FBS, and HbA1c level were recorded at the baseline. HbA1c level, fasting blood sugar, and weight were assessed at baseline and 90 days after enrolment, which is the end of the study. The primary endpoint in the study was HbA1c (%).

## Methodology

### Study population and recruitment criteria

SDRP is a retrospective study involving 150 patients with type 2 diabetes who participated in the program for 90 days and were adherent to program protocols. SDRP followed the ADA specified criteria for type 2 diabetes diagnosis for inclusion (HbA1c of 6.5% or over) and exclusion (HbA1c levels > 14%) of the participants [9]. Adults having type 2 diabetes with HbA1c > 6.5% done between March and September of 2021 and in the age groups of 20 to 80 years, were recruited to participate in SDRP from 2 metropolitan cities of India namely Bangalore and Delhi. Participants with HbA1c level > 14%, with diabetic complications involving vital organs, with associated comorbidities requiring tertiary support, and and the ones who did not adhere to the program protocols, were excluded from the study. A control or comparison group involved 110 individuals with type 2 diabetes on conventional pharmacotherapy not participating in the SDRP.

### SDRP program

SDRP is a personalized intervention program that uses technology-enabled medical management, dedicated coach-led diabetes, and nutrition experts to provide customized nutrition, progressive fitness, and behavioural modification for the holistic management of Type 2 diabetes.

Following recruitment, participants underwent a comprehensive diagnostic check-up in the fastened state for 70+ parameters to establish baseline metrics along with HbA1c and FBS. Additionally, age, gender, and weight were also recorded for each participant. A personal team of diabetes specialist doctors and nutrition and fitness coaches’ assistance was also provided to each participant. Participants were given unlimited access to their coaches through the app and via telephone, and on-demand doctor consultations for the entire duration of the program.

#### Glucose monitoring

Post baseline parameter assessment, a Continuous Glucose Monitoring (CGM) sensor (Libre Pro CGM Diabetes Sensor, Abbott Diabetes Care Limited, OXON OX29 OYL UNITED KINGDOM) was given to each participant to record the glucose profiles. CGM was recorded daily for 14 days from baseline. Post CGM data collection, a glucometer (Dr.Morepen GLUCO ONE Blood Glucose Monitoring System, Morepen laboratories limited, Himachal Pradesh, India) was also given to the participants for self-monitoring of blood glucose (SMBG) for the remainder of 15 to 90-days of the programme. Based on various factors like baseline HbA1c levels, comorbidities, complications and extent of progression, 1, 2 and 6 point SMBG profiling was advised. From the data derived from CGM and glucose monitoring, a comprehensive plan focusing on nutrition, fitness, and lifestyle modification was created for each participant according to the American Diabetes Association’s, ideal approach for diabetes management [9].

#### Personalized nutrition

Dietary intake plays a major role in maintaining blood glucose levels and weight management; hence, making food choices that help achieve normal blood glucose levels and ideal body weight is important. In this context, in SDRP, each participant was assigned a trained personal nutrition coach to assess nutritional needs and create a meal plan personalized to the individual’s lifestyle, resources, and food preferences. The coaches provide the diet plan having the optimal nutrient combination concentrating on complex carbohydrates, low glycemic index, glycemic load, and gut-healthy foods. Through one- on-one nutritional counselling the participants were guided to avoid specific foods that cause blood glucose spikes and replace them with food that does not produce spikes. Based on the first 14 days of CGM and client’s comfort level, health coaches suggested changes. Iterations were made after closely monitoring SMBG. For those individuals with higher BMI a 500 cal deficit diet of the recommended calorie intake per day was suggested after considering the 24 hour recall data and BMR [basal metabolic rate] calculation from Harris-benedict equation. Calorie deficit was done case by case and progressively depending on the client’s adherence to the plan.

#### Progressive fitness

Exercise training is recognized as a key factor in preventing and treating type 2 diabetes. The American College of Sports Medicine (ACSM) and American Diabetes Association (ADA) recommend moderate-intensity aerobic exercises and resistance training at least twice a week are beneficial for adults of all ages [10]. In alignment with this, in SDRP, each participant was assigned a diabetes expert coach to identify the areas of concern and develop a fitness routine. The fitness routine included brisk walks, yoga, and resistance training based on an individual’s fitness level and the desired goal. Goal for every participant was to achieve 10 k [10,000 steps] per day. In individuals with no movement challenges, clients were gradually given an additional 1000 step intervention from their baseline step count till they reached 10 k [timeline was not definite]. Ten minutes of walking post all 3 major meals everyday was suggested for all participants but it was never the only recommendation. Users were suggested live recorded sessions from the app and personal interactive sessions with the trainer on strength and conditioning and yoga. The coaches encouraged the participants to be physically active rather than sedentary during one-on-one sessions and ensured consistent participation.

#### Lifestyle modification

Lifestyle modification is a fundamental aspect of managing type 2 diabetes that includes self-management through behavioural change, mindfulness, and improved sleep quality. These approaches are reported to be preventive and complementary interventions for reducing stress, anxiety, depression, and improving the sleep quality in people with type 2 diabetes [9, 11, 12]. With this focus in mind, in SDRP, the participants were assigned personalized health coaches. The health coaches provided behaviour change strategies and diabetes education through counselling to help participants achieve positive health outcomes and live healthier and happier lives. Overall, the SDRP is a team-based care and a person-centered approach, assisted by technology to deliver one-on-one guidance and personalized interventions. The participants and the coaches interacted through multiple modes like chat, WhatsApp, voice, and video calls throughout the 90-day program, which minimized the chances of non-adherence, poor insights, and unpredictable outcomes.

### Participant consent and institutional review board approval

The SDRP program protocol was reviewed and approved by RxDx Healthcare Pvt. Ltd., Bangalore, India. The ‘control’ group data was taken from ‘A retrospective data collection of HblAc, fasting blood sugar, weight, and medication history of type-2 diabetic patients at baseline and 3 months and was reviewed and approved by Sehgal Nursing Home Institutional Ethics Committee, Delhi, India. Informed consent was also taken from each participant.

### Statistical analysis

The results were reported as means and standard deviation (mean SD). Mean value change from baseline to 90-days after enrolment was assessed using paired sample t-tests to evaluate the significant changes in HbA1c level, FBS, and weight. Age stratification was done for every 15 years starting from 20 to 80 grouping the population to 4 categories. Data is represented as Mean ± SD. The statistical significance was set at *p* < 0.05.

## Results

A total of 150 patients were enrolled and adhered to SDRP for 90 days. The participants were categorized into age buckets. Among 150 participants, 17% were in the age group of 20-35 years (yr), 49% were between 36 and 50 yr, 26% were between 51 and 65 yr, and 8% were between 66 and 80 yr. The average age of the participants was 50 years.

### HaemoglobinA1c (HbA1c) level

**Fig. 1:**
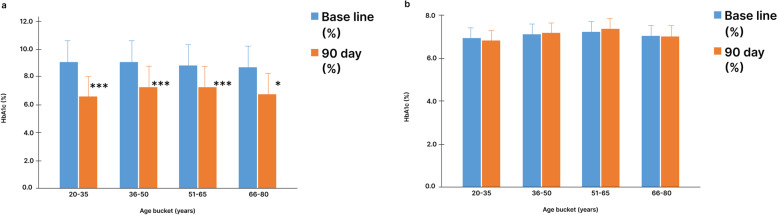
Change in mean Haemoglobin A1c level in study and control group participants from baseline to 90 days. **a** Haemoglobin A1c level in study group participants.** b** Haemoglobin A1c level in control group participants. Data is represented as Mean ± SD. HbA1c: Haemoglobin A1c**.** *: *p* < 0.05, ***: *p* < 0.001

The mean HbA1c level of the study group at baseline was 9.0 ± 1.5%. After 90 days into the intervention, it was reduced to 7.1 ± 1.3% with a mean HbA1c drop of 1.9 ± 1.5%. In SDRP, participants in the age group between 20 and 35 yr showed a drop of 2.5% in the HbA1c level from baseline, which is the highest drop compared to the rest of the age groups. Further, a lesser reduction of HbA1c level was shown in other age groups such as 1.8% in 36-50 yr, 1.5% in 51-65 yr and 1.9% in 66-80 yr (Fig. 1a). On the other hand, the control group had a mean HbA1c level of 7.13 ± 0.5% at the baseline and saw a slight increase to 7.18 ± 0.3% after 90 days of the study (Fig. 1b).

### Fasting blood sugar (FBS) level

**Fig. 2:**
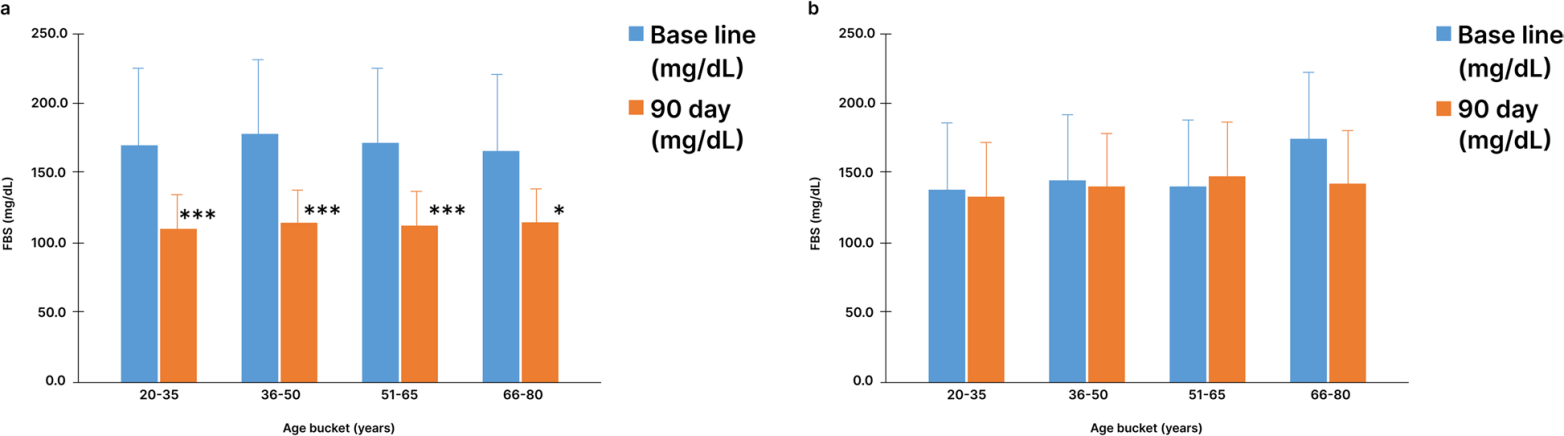
Change in mean fasting blood sugar level in study and control group participants from baseline to 90 days. **a** Fasting blood sugar level in study group participant.** b** Fasting blood sugar level in control group participants. Data is represented as Mean ± SD. FBS: Fasting blood sugar. *: *p* < 0.05, ***: *p* < 0.001

The study group had a mean FBS level of 178.3 ± 57.1 mg/dL at baseline. After 90 days into the intervention, it was reduced to 116.1 ± 24.4 mg/dL with a mean FBS drop of 62.2 ± 51.8 mg/dL. In SDRP, the highest reduction in FBS level was 65 mg/dL and this was seen in the age group of 36-50 yr. Rest of the other age groups showed similar reduction in FBS, and the observed drop was 61 mg/dL in 20-35 yr, 60 mg/dL in 51-65 yr and 53.5 mg/dL in 66-80 yr and (Fig. 2a). The control group, on the other hand, had a mean FBS level of 142.5 ± 48.1 mg/dL at baseline and decreased to 141.1 ± 44.8 mg/dL post 90 days, which is an insignificant drop of 1.42 ± 25.3 mg/dL (Fig. 2b).

### Body weight

**Fig. 3:**
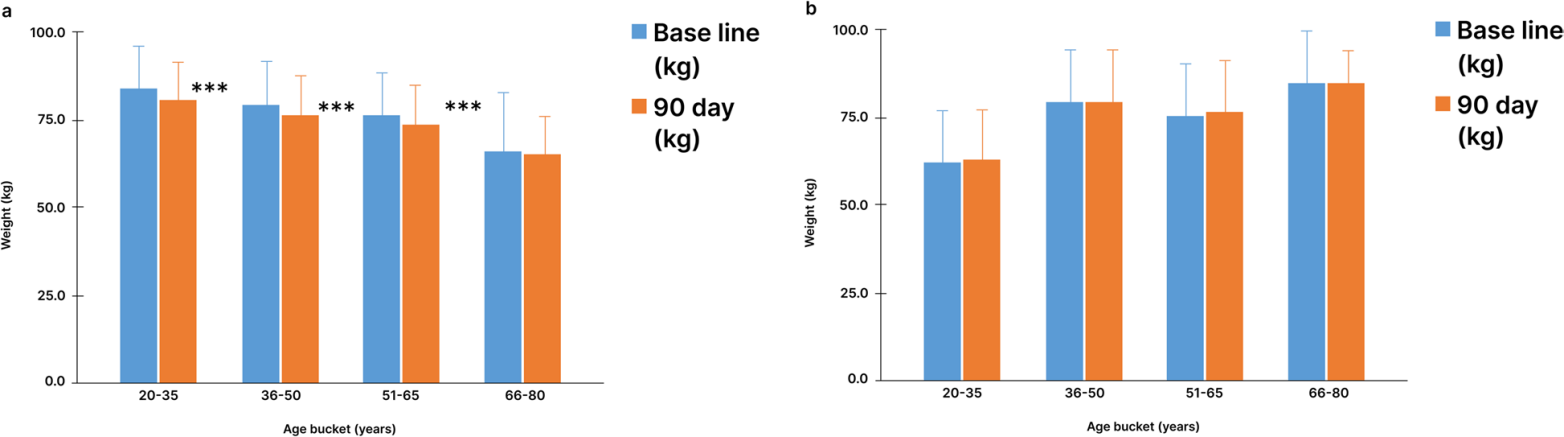
Change in mean weight of study and control group participants from baseline to 90 days. **a** Weight of study group participant**. b** Weight of control group participants. Data is represented as Mean ± SD. ***: *p* < 0.001

At baseline, the mean weight of the study group was 76.7 ± 12.7 kg, this decreased to 73.8 ± 11.8 kg with a mean loss of 2.8 kg after 90 days into the intervention. In SDRP, the weight loss was inversely proportional to the age of the participants. Participants in the age group of 20-35 yr showed the highest weight loss of 3.5 kg. There was also a significant weight loss among the different age groups, 3 kg in 36-50 yr, 2.5 kg in 51-65 yr and 1.4 kg in 66-80 yr (Fig. 3a). The control group, on the other hand, had a mean weight of 74.7 ± 9.6 kg at baseline and did not show significant weight loss after 90 days into the study (Fig. 3b). Infact, there was a slight gain of 0.5 kgs in weight of the control group from baseline to 90 days.

Overall the results indicated that the SDRP is an effective intervention for improving HbA1c, FBS, and weight in T2D and individuals younger than 20-35 yr showed better outcomes.

## Discussion

SDRP is a comprehensive, technology-enabled coach-led doctor-monitored program. Throughout the entire duration of study, health coaches collected data at set intervals about participants’ food intake, SMBG levels, HbA1c levels, physical activities, sleep quality, weight, height, and waist circumference. This data was used to develop tailored lifestyle interventions such as highly customized meal plans, progressive fitness regimens, and behavioural modifications for the holistic management of T2D. The study results showed that adhering to the holistic and individualized intervention designed in SDRP significantly improved the diabetes-related metrics- HbA1c, FBS, and weight within 90 days.

The SDRP led to a wide range of improvements in mean HbA1c levels compared to the control group. After 90 days, Hba1c reduction to < 7% was achieved for 16% of participants with A1c between 6.5-7.5, 29% between 7.5-8.5, 21% between 8.5-9.5%. Further, an A1c < 8% was seen in 21% of the participants with HbA1c between 9.5-11, and 13% with A1c above 11%. Additionally, 100% of participants achieved a mean FBS level of < 126 mg/dL, which is the cut-off for partial remission of diabetes [13]. In the weight loss category, 17% of the participants attained 4.3% loss, 49% attained 3.8% loss, 26% attained 3.3% loss, and 8% attained 2.1% loss indicating the improvements toward a suggested clinically significant weight loss of > 5% [9].

The study results demonstrated that SDRP is an effective intervention program and has shown marked improvements in all the various parameters that define T2D compared to the control group. The multimodal intervention approach comprising expert doctors, empathetic coaching for nutrition, progressive fitness, and mindfulness has led to a much greater impact on lowering A1c, FBS, and weight. The continuous glucose monitoring and a close follow up on parameters like SMBG, meals, weight, translates into personalized suggestions on food intake, exercise routine, and behavioural modifications having a significant impact on HbA1c levels and weight.

Previous studies have reported that personalized interventions concerning nutrition, physical activity, and behavioural modifications greatly impact HbA1c levels, glycaemic response, and weight management [14]. Kulzer et al. (2018) reported that an integrated, structured, and personalized approach has greatly benefited HbA1c reduction and maintenance [15]. Walker et al. (2015) described that lifestyle changes such as weight, diet, and physical activity contributes to the shift in glycaemic characteristics [16]. Further, studies have also reported that aerobic exercise, resistance training, and combined exercise improve the activation of the insulin signaling cascade, insulin sensitivity, body fat mass, thereby improving the overall metabolic health and reducing the metabolic risk factors, which greatly reduce the HbA1c level and benefit glycaemic controls in people with T2D [17–20]. Shamanna et al. (2020) also reported that adhering to a precision nutrition intervention helped people with T2D, decreasing the HbA1c, weight, and FBS within 90 days [21]. Further, the SDRP showed higher response in the younger age groups. The inter-individual variability could be contributed by the participants’ dietary habits, food behaviours, physical activity, phenotype, genotype, gut microbiota, etc., [14].

Various intervention studies have reported that a very low calorie, low carbohydrate, and low carbohydrate ketogenic diet decreases HbA1c levels, FBS, and reduces diabetes medications [4, 5, 22–24]. However, these studies focused majorly on the diet aspect only without attention to behavioral modification, which makes it difficult to adhere to these diets for a long term [25, 26]. Compared to these studies, the current study is more acceptable since it is a tailor-made multicomponent intervention plan focusing on diet, physical activity, and behavioural modifications for each individual yielding improvements in diabetes metrics within 90 days without any adverse health effects. It is reported that patients show a higher level of adherence to a personalized approach than the general recommendations [27]. A relatively small population and the short duration of the study were the limiting factors to validating the diabetes reversal. Few limitations of this study were, a) we do not have large population, especially older population, b) female subjects/population was less, c) month wise analysis for weight and fbs could have been included in the analysis. Thus, future studies should study the larger population and long-term effect of non-pharmacologic interventional approaches.

## Conclusions

The SDRP demonstrated that the reversal of type 2 diabetes is possible using a multi-interventional approach involving diabetic expert physicians and health coaches emphasizing personalized nutrition, physical activity, and behavioural modifications. The participants who adhered to SDRP for 90 days significantly improved their HbA1c level, FBS and weight compared to the control group who did not participate in the study. The study findings clarified that multiple components synergistically contributed to reducing the diabetic risk factors. Further, SDRP also demonstrated that adherence to non-pharmacologic interventional approaches is as essential as adherence to medication-only interventions. The study’s key strength was the personalization of the diet, exercise, and behavioural modifications.

## Data Availability

The datasets analyzed during the current study are available from the corresponding author on reasonable request.
